# FOXP3 and Tip60 Structural Interactions Relevant to IPEX Development Lead to Potential Therapeutics to Increase FOXP3 Dependent Suppressor T Cell Functions

**DOI:** 10.3389/fped.2021.607292

**Published:** 2021-02-03

**Authors:** Payal Grover, Peeyush N. Goel, Ciriaco A. Piccirillo, Mark I. Greene

**Affiliations:** ^1^Department of Pathology and Laboratory Medicine, Perelman School of Medicine, University of Pennsylvania, Philadelphia, PA, United States; ^2^Department of Microbiology and Immunology, McGill University, Montréal, QC, Canada; ^3^Program in Infectious Diseases and Immunology in Global Health, The Research Institute of the McGill University Health Centre, Montréal, QC, Canada; ^4^Centre of Excellence in Translational Immunology (CETI), Montréal, QC, Canada

**Keywords:** Foxp3, Treg—regulatory T cell, T effector cell, acetylation, histone acetyl transferase, TIP60, allosteric modifiers, IPEX

## Abstract

Regulatory T (Treg) cells play a role in the maintenance of immune homeostasis and are critical mediators of immune tolerance. The Forkhead box P3 (FOXP3) protein acts as a regulator for Treg development and function. Mutations in the *FOXP3* gene can lead to autoimmune diseases such as Immunodysregulation, polyendocrinopathy, enteropathy, and X-linked (IPEX) syndrome in humans, often resulting in death within the first 2 years of life and a scurfy like phenotype in *Foxp3* mutant mice. We discuss biochemical features of the FOXP3 ensemble including its regulation at various levels (epigenetic, transcriptional, and post-translational modifications) and molecular functions. The studies also highlight the interactions of FOXP3 and Tat-interacting protein 60 (Tip60), a principal histone acetylase enzyme that acetylates FOXP3 and functions as an essential subunit of the FOXP3 repression ensemble complex. Lastly, we have emphasized the role of allosteric modifiers that help stabilize FOXP3:Tip60 interactions and discuss targeting this interaction for the therapeutic manipulation of Treg activity.

## Immunodysregulation, Polyendocrinopathy, Enteropathy, and X-Linked (IPEX) Syndrome

IPEX is a rare and fatal disorder of immune dysregulation having an X-linked recessive pattern of inheritance. The first phenotype of IPEX was described in the early 1980s by *Powell et al*. when several male infants died within the first year of infancy due to diarrhea, hemolytic anemia, diabetes, thyroid dysfunction, eczema, and increased susceptibility to infections (1). IPEX is now defined by the clinical triad of autoimmune enteropathy, endocrinopathy (including neonatal type 1 diabetes and thyroiditis), and eczematous dermatitis. Due to the X-linked inheritance pattern, males are exclusively affected (IPEX can be lethal if left untreated within 2 years of infancy) while females are asymptomatic carriers and are healthy. IPEX is caused by mutations in the human *FOXP3* gene that normally influences the function of T regulatory (Treg) cells (2–4). It is interesting to mention that the intestine harboring a high frequency of Treg population is the most frequently affected organ in IPEX (5). The gastrointestinal tract is colonized by a plethora of microbial niches that provides an unparalleled challenge to the immune system. The latter responds effectively and efficiently for sustenance of active immune suppression against the microbial antigens eliciting an induced Treg development maintaining intestinal homeostasis (6, 7). However, disruption in Treg network such as mutations in *FOXP3* gene can thereby promote chronic intestinal inflammation. Various evidences from animal studies such as mouse models of ulcerative colitis/Inflammatory bowel disease indicate perturbation of Tregs promotes chronic intestinal inflammation to the intestinal microbiota (8). The early discovery of “scurfy” mice, a lymphoproliferative disease characterized by multi-organ lymphocytic infiltration was also linked to mutations in the *foxp3 gene* (9). This scurfy phenotype displayed homologous clinical and molecular features of IPEX in humans that were later mapped to the human orthologous *FOXP3* gene using sequence analysis (2, 3). The dynamic gut microbial dysbiosis and autoimmunity over the lifespan of scurfy mice have been demonstrated earlier (10).

**Figure 1:**
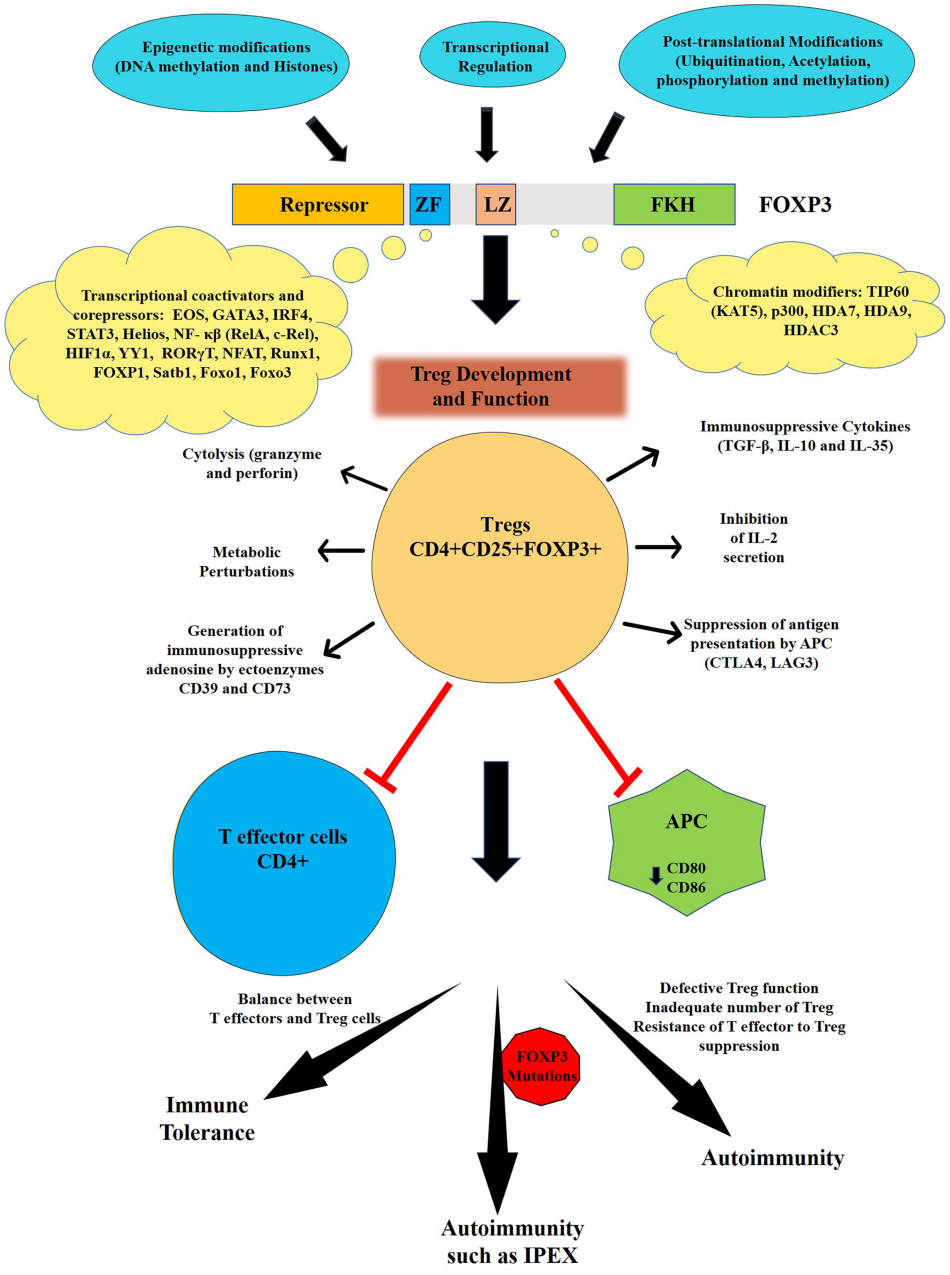
Regulation and function of FOXP3. The human FOXP3 has four distinct domains: N- terminal repressor domain, centrally located central zinc finger (ZF) and leucine zipper (LZ) and C-terminal Forkhead domain (FKH). The activity of FOXP3 is regulated by various epigenetic modifications, transcriptional, and post-translational modifications. FOXP3 is the master regulator for the development and function of regulatory T cells or Treg that are one of key mediators of immune tolerance and homeostasis. Tregs can suppress both arms of immune responses (innate and adaptive) through several mechanisms leading to maintenance of immune tolerance. Various mechanisms of Treg suppression events include secretion of immunosuppressive cytokines (TGF-β, IL-10, and IL-35); suppression by IL-2 consumption; induction of effector cell death via granzyme- and perforin-dependent cell cytolysis; production of immunosuppressive purine nucleoside adenosine by ectoenzymes CD39 and CD73; cAMP-mediated metabolic perturbation; downregulation of co-stimulatory molecules such as CD80 or CD86 on specialized Antigen presenting cells (APC) i.e., dendritic cells (DCs) via cytotoxic T-lymphocyte-associated protein 4 (CTLA4) and suppression of DCs via interaction of lymphocyte activation gene-3 protein (LAG3) present on Tregs with MHC-II on DCs. The breakdown of this tolerance leads to autoimmune diseases and auto-inflammatory events. Mutations in *FOXP3* gene, can lead to autoimmune condition such as IPEX in infants.

IPEX is one of the best characterized Mendelian disorders marked by the destruction of immune tolerance leading to autoimmunity owing to loss of functional CD4+CD25+ Treg cells (11–13) represented in Figure 1. Certain patients mimicking a similar phenotype but without *FOXP3* mutations are IPEX-like and is attributed to mutations in *CD25 (IL2RA), STAT1, STAT5b, CTLA4*, LPS-responsive beige-like anchor *(LRBA)*, Dedicator of cytokinesis 8 *(DOCK8) BACH2, ITCH*, and Mucosa-associated Lymphoid Tissue Lymphoma Translocation 1 (*MALT1*) genes (14–20). Many IPEX patients have significantly higher levels of serum IgE and more than half of such individuals display high levels of IgA while levels of IgM and IgG are in the normal range. Most patients have markedly decreased or absent FOXP3+ Treg cells, although some mutations do not significantly alter their cellular frequency in peripheral blood. Nonetheless, frequencies of both B and T lymphocyte cell subsets are normal in the majority of IPEX affected individuals (5–8, 12, 21).

The current therapeutic regimen for the treatment consists of supportive care (includes parenteral nutrition, blood transfusion, and insulin injections) followed by immunosuppressive therapy or bone hematopoietic stem cell transplantation (HSCT) (4, 12, 22). The immune-suppressive (IS) drugs influence the acute phase of autoimmunity in patients with IPEX syndrome. Various IS agents are used alone or as a part of multidrug regimens. These include glucocorticoids (prednisone or methylprednisolone and betamethasone) and steroid sparing agents such as calcineurin inhibitors (Tacrolimus and cyclosporin A) and sirolimus or rapamycin (23–25). Nevertheless, prolonged usage of IS agents may lead to opportunistic secondary infections. With the advancements in gene editing technologies, CRISPR-based therapy for IPEX syndrome was found to be promising under *in vitro* and *in vivo* settings (26). Moreover, chimeric antigen receptor (CAR)-modified Tcell gene therapies could be another blockbuster for treatment of genetic diseases including IPEX (27). However, in the current scenario, HSCT remains the most curative option for IPEX patients, but, potential complications such as graft-vs.-host disease (GVHD) and growth failure occur (28, 29).

## Forkhead Box P3 (FOXP3)

### Genetic Composition, Protein Structure, and Functional Analysis

FOXP3 belongs to the forkhead/winged-helix family of transcriptional regulators that includes 4 subfamily members FOXP1, FOXP2, FOXP3, and FOXP4, respectively. It is located on the X-chromosome (Xp11.23-Xq13.3) and is highly conserved in various species, including bovine, canine, murine and humans. There is almost 86% identical genetic sequence while 91% similarity in amino acid sequence between mouse and human counterparts (30, 31). Mutations in the *FOXP3* gene lead to IPEX in humans while a scurfy phenotype in mice (2, 9). To date, more than 70 distinct mutations have been reported in both the coding and non-coding regions of the *FOXP3* gene associated with IPEX (32). These include loss of function mutations, including missense, frameshift deletion, mutations in the polyadenylation site and mis-splicing (14).

In humans, *FOXP3* encodes a 431 amino acid (aa) containing protein with a molecular weight of 47.25 KDa. It consists of four distinct domains including the N-terminal proline rich domain responsible for transcriptional repression (from aa 1 to 193), the central zinc finger (ZF) (from aa 200 to 223) and leucine zipper (LZ) (from aa 240 to 261) that facilitates the homodimerization or heteromerization and the highly conserved C-terminal forkhead (FKH) domains (from aa 338 to 421) that enables nuclear localization and DNA binding activity (33–35) (representation shown in Figure 1).

The proline rich N-terminal domain in FOXP3 differs from the glutamine rich domain in other subfamily members and maybe recognized (36) for its distinctive role in the development and function of Tregs (37–39). FOXP3 acts as a “master” regulator of the Treg development process by interaction with various cofactors such as transcriptional and chromatin-modifying factors (40). It can act both as a transcriptional repressor or activator in Treg cells. In the thymus, a subset of CD4+CD25+ thymocytes can differentiate intra-thymically to develop into CD4+ FOXP3+ T cells known as natural (nTreg) or thymus derived (tTreg) cells. However, Treg cells can also develop from non-regulatory, conventional CD4+ T cells in peripheral lymphoid organs and non-lymphoid tissues through the action of cytokines like TGF-β, and IL-10. These CD4+FOXP3+ Tregs formed extra-thymically are referred to as induced (iTreg) (*in vitro*) or peripheral (pTreg) (*in vivo*) Treg cells (41, 42).

Initial reports documented the role of *Foxp3* as a transcriptional repressor (43). Co-transfection of *Foxp3* and a reporter gene containing a multimeric forkhead binding site demonstrated that Foxp3 could inhibit the transcription of the reporter. Furthermore, use of mutant protein lacking the key DNA-binding FKH domain, prevented nuclear localization, and failed to repress transcription. These results suggested that the repressive functions were dependent on the presence of the DNA binding FKH domain. In a subsequent study, using luciferase reporter plasmids containing binding sites for yeast Gal4 fused with FOXP3, it was established that the amino-terminal may be necessary for transcriptional repression (33).

We had shown that FOXP3 is a part of a complex consisting of the histone deacetylases HDAC7 and HDAC9, and the histone acetylase Tip60 (KAT5) that interact with its amino-terminal proline-rich domain (106–190 aa) of FOXP3 mediating transcriptional repression. Knockdown of endogenous Tip60 limited FOXP3-mediated repression while overexpression of Tip60 promoted suppression (36, 44, 45).

In another study on the suppressive function of FOXP3, AML1 (acute myeloid leukemia 1)/Runx1 (Runt-related transcription factor 1) which binds to FOXP3 in the region between the LZ and FKH domains also leads to upregulation of Treg-associated molecules by suppressing the expression and production of IL-2 and IFN-γ (46). FOXP3 interacts with nuclear factor of activated T cells (NFAT) to suppress the expression of inflammatory genes IL-2 and IFN-γ while functioning as a transcriptional activator of Treg cells by enhancing the gene expression of CD25, CTLA4, and glucocorticoid-induced TNF receptor (GITR) (47–50). In addition to the cofactors listed above, various other transcription factors have been reported to interact with FOXP3 serving as co-repressors or co-activators, respectively (40). Some of these include Interferon regulatory factor 4 (IRF4) (51), NF-κβ molecules RelA and c-Rel (52, 53), Retinoic acid receptor-related orphan receptor (RORγT) (54) RORα (55), Eos (IKZF4) (56), Helios (IKZF2) (57), hypoxia-inducible factor 1-alpha (HIF1α) (58), signal transducer and activator of transcription (STAT3) (59), YY1 (60), and GATA3 (61).

FOXP3 can form large molecular protein complexes that range from 300 KDa to more than 12000 KDa (36). Approximately 361 proteins potentially interact with FOXP3 directly or indirectly and are the part of an interactome as deduced by using biochemical and mass spectroscopy approaches (61). The functional significance of most of these interactions remains largely undefined. Using chromatin immunoprecipitation combined with genome-wide analysis, FOXP3 was thought to bind to approximately 700 different genes in Tregs and regulate them by either directly or indirectly activating or repressing them (50). The existence of these interactions highlights the master function of FOXP3 in Treg biology, but many of the expected regulatory events based on these proposed interactions have not been defined.

### Regulation of FOXP3 Expression

#### Epigenetic Modification

Epigenetic modification regulates the stability of FOXP3 expression via DNA methylation and histone modifications. The *FOXP3* promoter consists of various non-coding sequences CNS1, CNS2, CNS3 and CNS0 that are targets of modifying enzymes and subject to regulation at different stages of Treg development (62, 63). The CNS2 region is indispensable for Treg commitment and is required for FOXP3 expression in the progeny of dividing Treg cells and preventing autoimmunity (62). This region is rich in CpG motifs and DNA methylation studies have shown that these sites are highly demethylated within the *FOXP3* promoter of Treg cells, in turn, ensuring *FOXP3* mRNA transcription and lineage stability. tTreg cells are seemingly more stable in a functional sense than TGF-β-generated iTreg cells due to epigenetic modifications of *FOXP3*. *In vitro* antigenic stimulation of conventional CD4+ effector T cells in the presence of TGF-β leads to the expression of FOXP3 and acquisition of the suppressor phenotype (64). Surprisingly, these cells displayed no *FOXP3* DNA demethylation despite expression of FOXP3 whereas subsets of tTreg cells were stable even after expansion *in vitro* and remained demethylated. This demethylation of *FOXP3* CNS2 was exclusively associated with tTreg cells presenting FOXP3 as the most specific marker for tTreg and also provides an explanation as to why tTreg are more stable than TGF-β induced Treg cells (65, 66). The CpG demethylation or hypomethylation of CNS2 or Treg-specific demethylated region (TSDR) provides stability of FOXP3 while methylation or hypermethylation results in transient expression of FOXP3 occurring only in T effector cells (67). Also, Satb1, a transcriptional factor binds to CNS0 and functions as a chromatin organizer providing access for histone modifications (63). Satb1 is considered as a pioneer factor of Treg differentiation as its occurrence precedes FOXP3 in Treg precursor population while inhibiting Satb1 reduces FOXP3 levels and subsequent Treg development (63).

Histone modifications such as methylation and acetylation of H3 influences the Treg development by regulating FOXP3 expression (68, 69). In a recent study, MLL4, a histone methyltransferase that promotes mono-methylation of H3K4 by binding to FOXP3 promoter, was suggested to be a critical regulator of Treg cell development as deduced from studies using conditional deletion of *Mll4* in CD4+ T cells (70). Histone methyltransferase, SMYD3 regulates the expression of FOXP3 in iTreg cells by trimethylating histone H3K4 in the promoter and CNS1 region of *FOXP3*. However, the ablation of SMYD3 led to a reduction in H3K4me3 and the abrogation of suppressive capabilities of iTreg cells (71). Studies have also suggested that Histone deacetylase 3 (HDAC3) mediates the development and function of Tregs while conditional deletion of HDAC3 in a murine model blocked the suppressive function of Treg cells (72). Taken together, these findings ascertain the key role of epigenetic modifications in regulating FOXP3 expression and orchestrating Treg cell functional development.

#### Transcriptional Regulation

In addition to epigenetic modulation, an array of transcriptional events controls the transcription of FOXP3 depending on the signals T cells receive either in thymus or periphery (73, 74). Many transcription factors bind to CNS regions along with the *FOXP3* promoter during the Treg developmental process. Binding of transcription factors to the demethylated CNS2 is required for the heritable sustenance of FOXP3 and CNS3 for the induction of FOXP3 expression (62). Foxo transcription factors, primarily Foxo1 and Foxo3, induce FOXP3 expression by translocating to the nucleus and bind to CNS2 and promoter regions of *FOXP3*, and contribute to Treg commitment. These transcriptional factors are also characterized by a conserved winged helix DNA binding and are essential for defining the program of T cell differentiation especially into tTreg cells expressing FOXP3. However, upon T-cell receptor (TCR) activation by antigenic stimulation, phosphatidylinositol 3-kinase (PI3K)/protein kinase B (Akt) pathway gets activated and Akt phosphorylates Foxo1/Foxo3 that leads to their inactivation and nuclear exclusion (75–78).

One NF-κB transcription family member, c-Rel, also plays a crucial role in tTreg cells by binding to the promoter, CNS2 and CNS3 regions of *FOXP3* allowing its active transcription (79, 80). A previous study demonstrated that c-Rel promotes FOXP3 expression by the formation of an enhanceosome containing c-Rel, p65, NFAT, Smad, and CREB promotes FOXP3 expression in tTreg cells (81). In pTreg cells, both Smad2 and Smad3 are critical for TGF-β-mediated Foxp3 induction. TGF-β signaling triggers the activation of both these transcription factors Smad2/3 that bind to enhancer regions of the *foxp3* elevating FoxP3 expression in pTreg cells (82, 83). Our laboratory established that both Smad3 and NFAT are required for histone acetylation in this enhancer region for the induction of Foxp3 (83). Other members of the NF-κB and Nr4a family also contribute to the induction of FOXP3 in pTreg cells (80, 81, 84). These findings validate the fine tuning of Treg cells development, intra-thymically or extra-thymically orchestrated by different transcriptional mechanisms that modulate *FOXP3* transcription.

#### Post-translation Modifications

The expression of FOXP3 can also be regulated at the protein level by various post-translational modifications (PTMs) such as phosphorylation, ubiquitination, methylation, and acetylation (85). These modifications influence the stability, DNA binding capacity, subcellular localization, protein interactions (transcriptional activators/repressors and chromatin Remodelers) of FOXP3, all events which collectively contribute to the induction or maintenance of Treg function.

##### Phosphorylation

The phosphorylation of FOXP3 can occur on serine, threonine, and tyrosine residues. We have been the first to identify that chromatin bound FOXP3 can be phosphorylated at threonine residues (86). Since then, several reports have identified various kinases that phosphorylate FOXP3 and depending on the site, can either activate or inhibit FOXP3. The N-terminal repressor domain of FOXP3 consists of 4 cyclin dependent (CDK) motifs (Serine/Threonine-Proline) that can be phosphorylated by CDK2/cyclin E. This phosphorylation leads to a reduction in Treg suppressive activity and thus negatively regulates Treg cellular function. However, mutation of Serine/Threonine to Alanine at each CDK motif significantly increased FOXP3 stability and Treg suppressive ability (87). Pim-1 and Pim-2, oncogenic serine/threonine kinases can also phosphorylate FOXP3 and negatively regulate the Treg suppressive function (88, 89).

Pim-1 phosphorylates Ser422 located in the FKH domain and limits binding of FOXP3 to its target genes lessening its transcriptional activity. However, inhibition of Pim-1 improved FOXP3 DNA binding activity and enhanced the suppressive activity of Treg cells (88). Pim-2 phosphorylates at multiple sites in the N-terminal domain of FOXP3 contributing to decreased suppressive functions of Treg by altering the expression of cell-associated surface markers, including CD25 and GITR (89). In contrast, certain kinases can also induce FOXP3 expression. Lymphocyte-specific protein tyrosine kinase (LCK) phosphorylates Tyrosine-342 of FOXP3 and upregulates its expression resulting in decreased Matrix metalloproteinase 9 (MMP9), S-phase kinase-associated protein 2 (SKP2), and Vascular endothelial growth factor A (VEGF-A) levels in cancer cells (90). More recently, it was shown that Nemo-like kinase (NLK) can phosphorylate FOXP3 on multiple residues and stabilize it by preventing ubiquitin-mediated proteasomal degradation and thus contribute to sustained Treg suppressive function. However, the conditional deletion of NLK causes loss of Treg suppressive activity and increased inflammation (91).

##### Methylation

The activity of FOXP3 can be regulated by methylation on arginine residues by Protein arginine methyltransferases PRMT1 and PRMT5. PRMT1 methylated Arginine48 and Arginine51 upon interaction with FOXP3. This was confirmed using selective inhibitors against PRMT1 that attenuated the Treg suppressive functions. Further, the activity of a methylation defective mutant of FOXP3 was sharply reduced in curbing graft-vs.-host disease *in vivo* (92). Additionally, our laboratory demonstrated that PRMT5 can methylate FOXP3 and conditional deletion of PRMT5 in Tregs modulate their numbers and function resulting in a scurfy-like phenotype *in vivo* (93).

##### Ubiquitination

Ubiquitination is one of the most extensively studied regulatory modifications. Protein ubiquitination is a multistep process that involves three different enzymes i.e., E1 or ubiquitin activating enzyme, E2 or ubiquitin conjugating and E3 or ubiquitin ligase. The ubiquitin polypeptide consists of 7 lysine residues and each of them participates in its own poly-ubiquitin chain (94). Ubiquitination of FOXP3 is related to lysine48 type polyubiquitination that marks it for 26S proteasome-mediated degradation (95). FOXP3 typically undergoes proteasomal degradation under stress conditions. For instance, in a hypoxic environment, levels of Hypoxia-inducible factor-1, also known as HIF-1α are upregulated. The latter binds to FOXP3 and targets it for proteasomal degradation, thereby attenuating Treg development (58).

Streptococcus infections in psoriasis patients lead to the induction of CCL3 that decreased FOXP3 stability by promoting its degradation (96). Stub1, an E3 ubiquitin ligase promotes lysine48 polyubiquitination of FOXP3 in an Hsp70-dependent manner in response to an inflammatory stimulus raised by proinflammatory cytokines and lipopolysaccharide (LPS). Knockdown of Stub1 prevented FOXP3 degradation while overexpression in Treg cells abolished their capacity to repress inflammatory immune responses *in vitro* and *in vivo* (97). In contrast, deubiquitinase enzyme (DUB) USP7 is overexpressed in Tregs where it associates with FOXP3 in the nucleus and regulates FOXP3 turnover. The knockdown of USP7 decreased the levels of endogenous FOXP3 and diminished the Treg suppressive activity while ectopic expression of USP7 increased FOXP3 expression by preventing its degradation (98).

##### Acetylation

The activity of FOXP3 can be regulated by acetylation and deacetylation of specific lysine residues. Using mass spectroscopy and later structure-guided mutagenesis, various lysine residues (31, 262, 267, 250, and 252) in FOXP3 subject to acetylation were identified (99, 100). Acetylation augments the stability of FOXP3 and increases its DNA binding ability leading to elevated Treg suppressive function by facilitating the binding of FOXP3 to its transcriptional targets. The processes of both acetylation and ubiquitination target lysine residues and thus compete where ubiquitination targets FOXP3 for proteasomal mediated protein degradation while acetylation imparts stability to FOXP3 and prevent it from degradation (101).

The process of acetylation and deacetylation is catalyzed by Histone acetyltransferases (HATs)/lysine acetyltransferases (KATs) and Histone deacetylases (HDACs). Both Tip60 (KAT5), a member of MYST family and p300 (KAT3b), a member of p300/CBP family, are the major acetylases that acetylate FOXP3 enhancing DNA binding and stability of FOXP3 (44, 101, 102). We have shown that both Tip60 and p300 positively regulate FOXP3 acetylation in a cooperative manner and provided a proof of concept of the vital role of Tip60 in maintaining peripheral Treg cell population that keeps a check on limiting autoimmune responses (103). In contrast, Sirtuin 1 (SIRT1), a member of the lysine deacetylase co-localizes with Foxp3 in the nucleus and results in decreased acetylation of FOXP3. Further, inhibition of SIRT1 decreases poly-ubiquitination of FOXP3, thereby increasing FOXP3 protein levels (104). FOXP3 also associates with Tip60, HDAC7, and HDAC9 at the N-terminal to form a chromatin remodeling complex, which alters the acetylation state of FOXP3 (45).

### Effect of Interactions of Tip60 With FOXP3 and Precision Therapy

The first report that described that activity of FOXP3 is regulated by Tip60-mediated acetylation came from our laboratory (44). Tip60, HDAC7, and HDAC9 were associated in a dynamic ensemble with FOXP3, and Tip60 was found to function as the essential subunit of FOXP3 repression complex. The N-terminal of FOXP3 106–190 aa of the repressor domain is required for Tip60-FOXP3 interactions. Further, overexpression of Tip60 but not its HAT-deficient mutants promoted FOXP3 mediated repression while knockdown of endogenous Tip60 alleviated the transcriptional repression (44). Our study indicates that Treg function can be modulated by acetylation of FOXP3 toward a suppressive phenotype and can be subjected to therapeutic interventions such as altering of enzymatic activity of HATs or HDAC to modify Treg functions.

**Figure 2:**
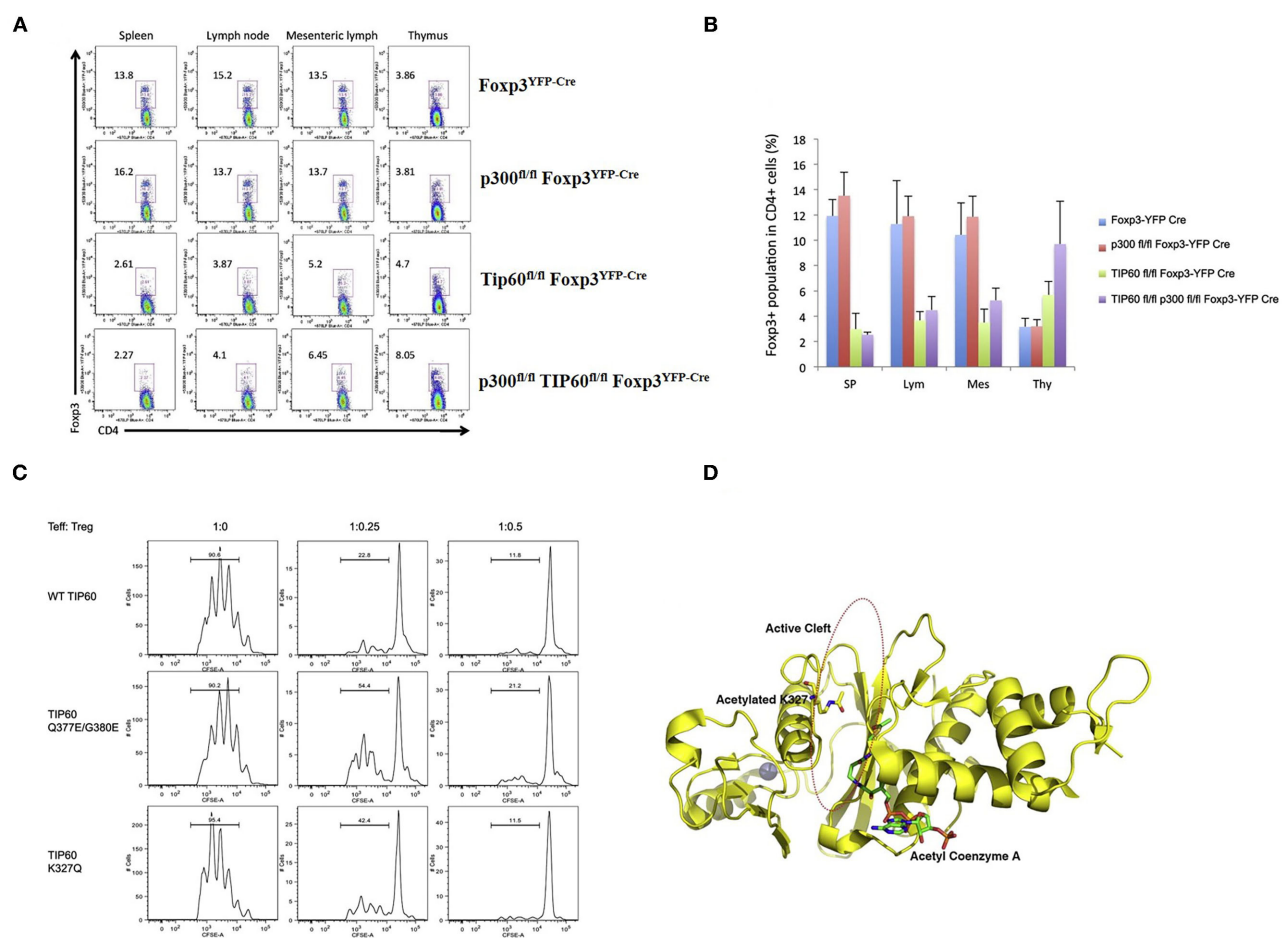
Role of Tip60 in maintenance of peripheral Treg cell [Figure adapted from (103)]. **(A)** Dot plots of Treg cell populations from Foxp3^YFP−Cre^, p300^fl/fl^ Foxp3^YFP−Cre^, Tip60^fl/fl^ Foxp3^YFP−Cre^, and p300^fl/fl^ Tip60^fl/fl^ Foxp3^YFP−Cre^ mice. **(B)** Average percentage of Foxp3 expressing CD4+ T cells in spleen, lymph nodes, mesenteric lymph, and thymus. **(C)** CD4+ T cells transduced with both Foxp3 and WT Tip60 or Tip60 mutants (Q377/G380E and K327Q) and assessment of the suppressive function of transduced cells. **(D)** Structure model of Tip60 (acetylated at K327) favoring Foxp3 binding at cleft.

After the initial discovery of FOXP3 acetylation by our laboratory, another HAT, p300 was discovered that can acetylate FOXP3 and regulate Treg cells. Acetylation of FOXP3 is reciprocally regulated by the HAT p300 and HDAC SIRT1 (101). In a subsequent study, data from our laboratory demonstrated that both Tip60 and p300 can cooperatively acetylate FOXP3 (103). We proposed that the interaction of p300 with Tip60 boosts autoacetylation and improves the stability of Tip60. Following this activation of Tip60, p300 acetylates K327 of Tip60, which then acts as a molecular switch facilitating Tip60 to change binding partners. This event releases p300 from Tip60 and consequently Tip60 associates with another substrate, FOXP3, that it later acetylates. Inversely, Tip60 also promotes p300 acetylation critical for its HAT activity. Synergistic interactions of Tip60 and p300 lead to the maximal induction of FOXP3 activity. Interestingly, conditional knockout of Tip60 but not p300 in Treg cells *in vivo* reduced Treg population significantly in peripheral organs, leading to catastrophic scurfy like disease (103). Notably, knockout of Tip60 in Foxp3 expressing cells decreased the Treg cell population in both spleen and lymph nodes while displaying a differential effect in thymus or periphery. Finally, CD4+ naïve T-cells transduced with both Foxp3 and wild type (WT) Tip60 or Tip60 mutants (Q377/G380E and K327Q) demonstrated decreased suppressive function compared to WT Tip60 [Figure 2 adapted from (103)]. These results clearly represent the indispensable role of Tip60 in maintenance of peripheral Treg cells.

The importance of FOXP3 in regulating Tregs is supported by a study in which patients with rheumatoid arthritis, a chronic autoimmune disease exhibited an insufficient expression of FOXP3 inducing a deficiency in Tregs. Mechanistically, it was shown to be related to inadequate levels of Tip60. Ectopic expression of Tip60 restored FOXP3 levels, directing Treg differentiation and its suppressive activity in a model of rheumatoid arthritis (105).

**Figure 3:**
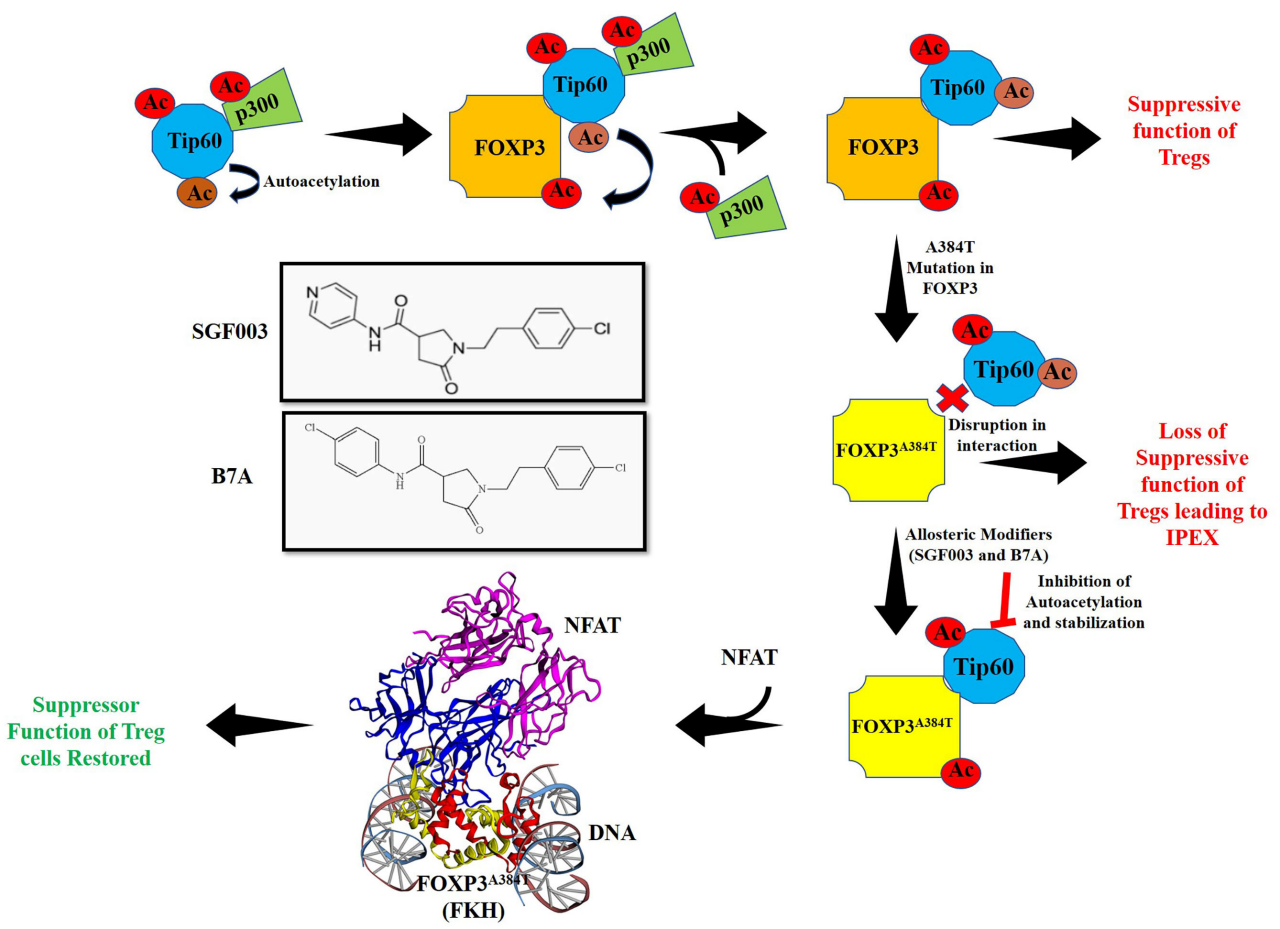
Acetylation of FOXP3 by cooperative interactions between Tip60/p300 and hypothetical model for action of allosteric modifier for Treg cell function. Interaction of HATs Tip60 and p300 leads to autoacetylation of Tip60 and p300 mediated acetylation of Tip60 at K327. Inversely, Tip60 also acetylates p300. The result of these cooperative interactions acts as a molecular switch that releases p300 ensuing Tip60 interacts with FOXP3 and subsequently acetylating it. In autoimmune disorders such as IPEX, mutations in *FOXP3* lead to dysregulation of immune homeostasis by disrupting the function of Tregs. One of the major mutations A384T in *FOXP3* leads to disruption of Tip60:FOXP3 interaction. Use of allosteric modifiers SGF003 and B7A can, however stabilize these interactions by inhibiting autoacetylation of Tip60 and delaying release of Tip60 from the complex. This leads to correction of suppression and restoration of Treg suppressive function. The allosteric modifier B7A inhibits Tip60 autoacetylation and stabilizes its interaction with FOXP3^A384T^. This allows access of NFAT to interact with FOXP3^A384T^ forming a stabilized complex leading to restoration of Treg cell function. Here, the crystal structure with PDB ID 3QRF (108) of FOXP3 FKH domain (red and yellow) complexed with DNA and NFAT (blue and purple) using EzMol (109) is represented.

One of the most common mutations in IPEX, Alanine to Threonine (A384T), occurs in the FKH domain of FOXP3. This mutation can explicitly disrupt the FOXP3-Tip60 interactions affecting Treg function. The mutation permits some level of FOXP3 DNA binding capacity and the ability of Treg to suppress inflammatory cytokine production (3, 106). We identified small allosteric molecules that target Tip60 using a cavity induced allosteric modification (CIAM) approach (106, 107). These synthetic allosteric modifiers (SGF003 and B7A) can help stabilize Tip60-FOXP3 interactions and promote Treg functionalities. Mechanistically, these partially inhibit the autoacetylation of Tip60, thus delaying the release of Tip60 from Tip60-p300-FOXP3 complex. Hence, loss of function could be rescued to increase Tip60-Foxp3 interactions by using such a pharmacological intervention (Figure 3). In a recent finding from our group, treatment with SGF003 or B7A led to a rapid and marked increase in Tip60 binding to both WT and A384T FOXP3 (106).

Furthermore, we have also shown that both compounds heightened the suppressive capacity of murine and human Tregs without affecting the proliferation, capacity to secrete inflammatory cytokines such as IFN-γ or Th17 differentiation of murine and human responder T effector cells. Therapeutic potential of the Tip60 modifier was studied in a dextran sodium sulfate (DSS) induced colitis model and collagen-induced arthritis (CIA) model, respectively. The allosteric modifier B7A was able to provide protection in both the disease models by promoting Treg function without directly affecting T effector cellular responses and thus significantly improving autoimmune symptoms (106).

In a more recent study, the interaction of FOXP3-NFAT was assessed in the presence of B7A in both WT and A384T FOXP3 mutants, and we observed that B7A enhanced the interaction of FOXP3 with NFAT in both of WT and A384T (93, 106). The hypothetical model of FOXP3^A384T^-NFAT interaction is represented in Figure 3. Taken together, these results identify the therapeutic potential of Tip60 allosteric modifiers in autoimmune diseases. Moreover, the development of higher affinity forms of these allosteric modifiers might provide prospective opportunities for targeting Tregs in various disease conditions.

## Author Contributions

All authors listed have made a substantial, direct and intellectual contribution to the work, and approved it for publication.

## Conflict of Interest

The authors declare that the research was conducted in the absence of any commercial or financial relationships that could be construed as a potential conflict of interest.
